# A molecular understanding of alphavirus entry

**DOI:** 10.1371/journal.ppat.1008876

**Published:** 2020-10-22

**Authors:** Autumn C. Holmes, Katherine Basore, Daved H. Fremont, Michael S. Diamond

**Affiliations:** 1 Department of Medicine, Washington University School of Medicine, St. Louis, Missouri, United States of America; 2 Department of Pathology and Immunology, Washington University School of Medicine, St. Louis, Missouri, United States of America; 3 Department of Molecular Microbiology, Washington University School of Medicine, St. Louis, Missouri, United States of America; 4 Department of Biochemistry and Molecular Biophysics, Washington University School of Medicine, St. Louis, Missouri, United States of America; 5 The Andrew M. and Jane M. Bursky Center for Human Immunology and Immunotherapy Programs, Washington University School of Medicine, St. Louis, Missouri, United States of America; NYU Langone Health, UNITED STATES

## Abstract

Alphaviruses cause severe human illnesses including persistent arthritis and fatal encephalitis. As alphavirus entry into target cells is the first step in infection, intensive research efforts have focused on elucidating aspects of this pathway, including attachment, internalization, and fusion. Herein, we review recent developments in the molecular understanding of alphavirus entry both in vitro and in vivo and how these advances might enable the design of therapeutics targeting this critical step in the alphavirus life cycle.

## Introduction

Alphaviruses are enveloped, positive-sense, single-stranded RNA viruses in the *Togaviridae* family that are transmitted by arthropods and are responsible for emerging and reemerging diseases in humans. Some alphaviruses (e.g., Chikungunya (CHIKV), Ross River (RRV), Mayaro (MAYV), Semliki Forest (SFV), Sindbis (SINV), and O'nyong-nyong (ONNV)) cause acute inflammatory musculoskeletal and joint-associated syndromes, which can become chronic [1], whereas others (Eastern (EEEV), Western (WEEV), and Venezuelan (VEEV) equine encephalitis viruses) cause infection in the brain and neurological disease (Table 1). Although pathogenic alphaviruses are maintained in sylvatic transmission cycles in nature, their insect vectors and reservoir host species vary, which has implications for their geographic range and potential for causing outbreaks in humans.

**Table 1:** Diseases of pathogenic alphaviruses, mosquito vectors, and reservoir hosts.

Medically relevant alphavirus	Antigenic clade	Confirmed or putative mosquito vector(s) species	Confirmed or putative reservoir host(s)	Disease manifestations
CHIKV	SFV	*Aedes albopictus* [128], *Aedes aegypti*, *Aedes* (subgenus *Stegomyia*)	Nonhuman primates [1]	Fever, polyarthralgia (frequently becomes recurrent), myalgia, rash, and headache
ONNV	SFV	*Anopheles funestus*, *Anopheles gambiae* [129]	Unknown	Similar to CHIKV with the addition of cervical lymphadenitis
MAYV	SFV	*Haemagogus janthinomys* [130], *A*. *aegypti* [131]	Nonhuman primates [132]	Identical to CHIKV
RRV	SFV	*Culex annulirostris*, *Aedes vigilax* [133]	Marsupials [134]	Identical to CHIKV
SFV	SFV	*Aedes* spp. [135]	Small mammals, birds, nonhuman primates [135]	Mild febrile illness in humans; infrequent myalgia and polyarthralgia; encephalitis can be induced in mice
EEEV	EEEV	*Culiseta melanura* [136], *Culex erraticus* [137]	Passeriformes birds [136]	Similar to CHIKV if there is no CNS involvement; encephalitic disease includes headache, vomiting, diarrhea, seizures, and coma
SINV	WEEV	*Culex* spp. [138]	Wild birds [139]	Arthralgia, rash, malaise
VEEV	VEEV	*Culex* (Melanoconion) spp. [140]	Small mammals [132]	Similar to EEEV; infection has lower mortality rate than EEEV
WEEV	WEEV	*Culex tarsalis* [141]	Wild birds [141]	Mainly subclinical or nonspecific febrile illness; can progress to encephalitis in rare cases

Listed are the alphaviruses most frequently associated with disease outbreaks in humans. The reservoir hosts for these viruses include nonhuman primates, rodents, birds, and marsupials. Humans and equines represent either accidental hosts or are involved in epizootic transmission cycles. Mosquitoes from the *Aedes* and *Culex* genera are the major vectors of pathogenic alphaviruses, and at least 30 different species have been implicated.

CHIKV, Chikungunya; CNS, central nervous system; EEEV, Eastern equine encephalitis virus; MAYV, Mayaro; ONNV, O'nyong-nyong; RRV, Ross River; SFV, Semliki Forest; SINV, Sindbis; VEEV, Venezuelan equine encephalitis virus; WEEV, Western equine encephalitis virus.

**Fig 1:**
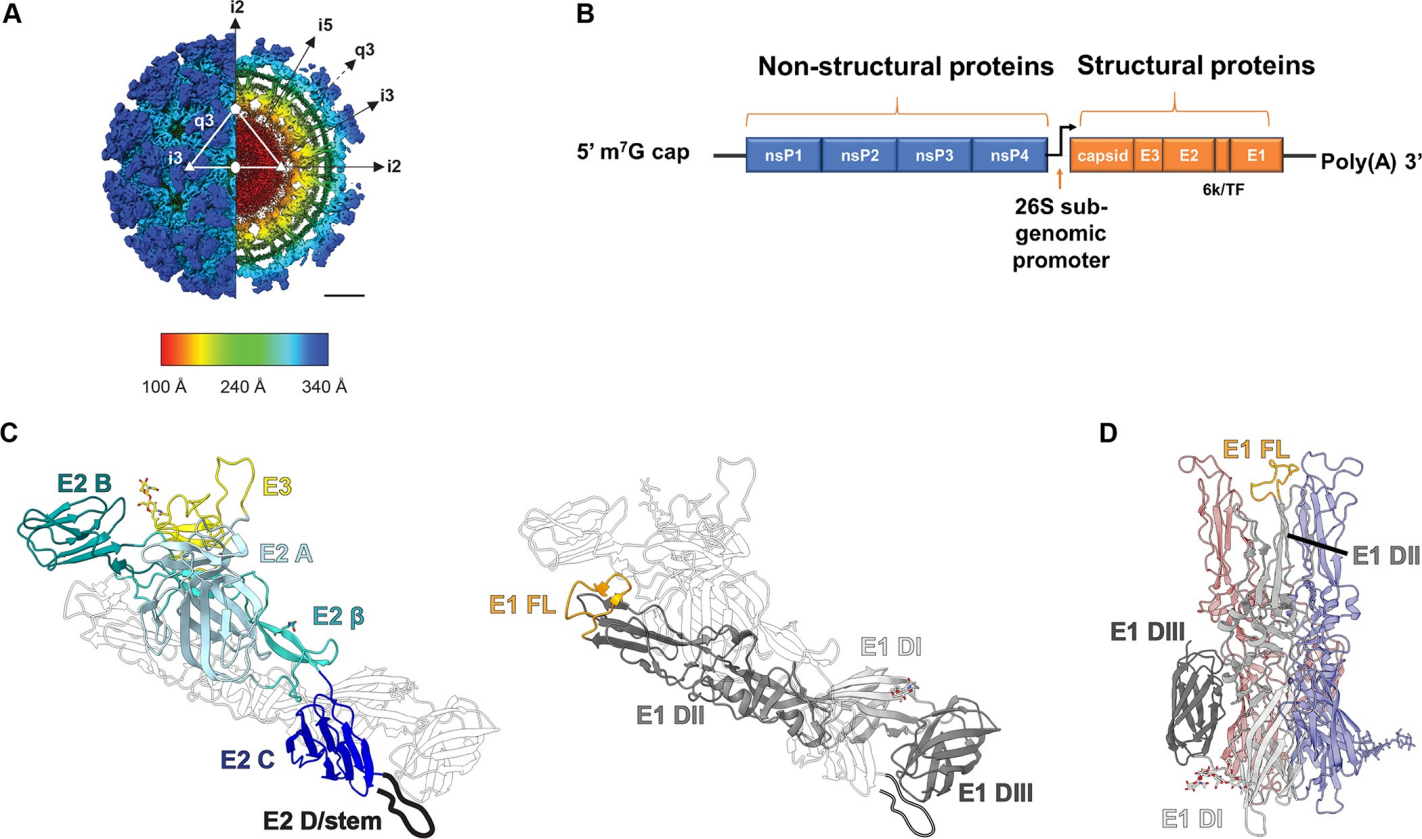
Alphavirus genome organization and molecular structure of the virion. **(A)** Cryo-EM reconstruction of CHIKV VLP (EMDB: 9393) colored by radial distance and depicted from the surface (left half) and an equatorial cross section (right half). The white triangle indicates 1 icosahedral asymmetric unit, with the 5-fold (i5), 3-fold (i3), and 2-fold (i2) icosahedral axes of symmetry labeled with a pentagon, triangles, and an oval, respectively. Trimeric spikes are labeled “i3” if coincident with the i3 axes and “q3” if on a quasi-3-fold axis. The black arrows indicate the directions of the icosahedral symmetry axes. Radial distance color scheme: red, electron dense core and RNA; yellow, capsid; green, membrane lipid; cyan, E1; and dark blue, E2 spike. Scale bar: 100 A°. E1 and E2 are embedded in the viral membrane and assemble into a heterotrimer: E1 is responsible for membrane fusion, while E2 facilitates receptor engagement. E3 is also bound in some alphaviruses including CHIKV, SFV, and VEEV, but the significance of this is not fully understood. **(B)** The alphavirus genome is a single-stranded, plus-sense RNA molecule of approximately 11 kb and encodes 4 nonstructural proteins, nsP1–4 and 5 structural proteins, capsid, E3, E2, 6k/TF, and E1. NsP4 forms the primary RNA-dependent RNA polymerase, but the synthesis of the genome requires all 4 nonstructural proteins. The RNA is capped at the 5′ end and polyadenylated at the 3′ end. **(C)** The alphavirus structural proteins E2 and E3 are produced as a polyprotein termed p62 (left). P62 acts as a chaperone to ensure proper folding of E1 (right) in the ER and is proteolytically processed into the mature E2 and E3 proteins by host furin-like proteases. E3 binds E2 during transport of the protein complex to the cell surface and also remains bound in the mature virion for some alphaviruses. Structural image generated with Chimera software using structural data reported in [11] (PDB:3N40). Both glycoproteins are colored by domain. E3, yellow. E2: domain A, light blue; β-ribbon connector, light cyan; domain B, dark cyan; domain C, dark blue; cartoon of subdomain D/stem region, black. E1: DI, light gray; DII, medium gray; DIII, dark gray; FL, orange. **(D)** Upon exposure to low pH, E2 dissociates from E1, which drives E1 homotrimer formation (structural representation pictured). The FLs are exposed, which insert into the target membrane and facilitate membrane fusion in the early endosome. Image generated with Chimera software and structural data reported in [34] (PDB:1RER). One of the 3 E1 monomers is colored by domain. CHIKV, Chikungunya; cryo-EM, cryo-electron microscopy; ER, endoplasmic reticulum; FL, fusion loop; SFV, Semliki Forest; TF, transframe; VEEV, Venezuelan equine encephalitis virus; VLP, virus-like particle.

The alphavirus virion is approximately 70 nanometers in diameter and has T = 4 icosahedral symmetry (Fig 1A) [2,3]. The spherical virion is comprised of a single approximately 11.4 kb RNA genome encapsidated in a nucleocapsid core and surrounded by a host-derived lipid membrane. The genome encodes 4 nonstructural proteins, nsP1–4, which mediate viral translation, viral replication, and host subversion and evasion [4] and 6 structural proteins, capsid, E3, E2, 6K, transframe (TF), and E1 (Fig 1B). E1 and E2 are transmembrane proteins that interact to form a heterodimer (Fig 1C). Trimers of E1/E2 heterodimers assemble into higher order spikes (80 in total) on the virion surface. The alphavirus E2 protein facilitates receptor engagement [5], whereas E1 principally mediates membrane fusion after viral entry [5,6]. The carboxyl terminus of E2 also interacts with the capsid core, which stabilizes the virion [7,8]. The 6K protein is thought to promote glycoprotein maturation, spike assembly, and act as a viroporin [9]. The 6K gene produces 2 proteins, 6K and TF, the latter of which also contributes to virus particle assembly [9]. The TF product associates with E1/E2 and is detected on the virion surface, albeit at lower stoichiometric levels than other structural proteins [5]. TF also inhibits type I interferon (IFN) responses in cultured cells and in vivo through a mechanism dependent upon palmitoylation of the protein [10].

Over the past several decades, many groups have investigated the steps of alphavirus cellular entry given its implications for tropism. In this review, we summarize our understanding of alphavirus attachment and entry, as revealed by recent structural, biochemical, and genetic studies. We discuss how recent advances could be harnessed for possible therapeutic intervention.

## Alphavirus structural proteins and their involvement in entry

### E1/E2 structure and function

The E2 glycoprotein is translated in the infected cell in conjunction with E3 as a polyprotein termed p62, also called PE2. P62 co-translationally associates with E1 (Fig 1C) within the endoplasmic reticulum (ER), an interaction that is required for proper folding of E1 [11]. Subsequently, p62 is processed into the mature E2 and E3 proteins by furin-like proteases in the trans-Golgi network [12]. After furin cleavage, E3 remains associated with E2 at acidic pH to stabilize the heterodimer and prevent premature fusion within secretory vesicles [13–16]. For most alphaviruses, E3 dissociates from the virion in the neutral pH environment of the extracellular space. This coordinated binding and dissociation of E3 ensures the generation of a fusion-competent, infectious particle. Indeed, when the furin cleavage site of p62 is mutagenized, the resultant virion is less infectious and requires a lower pH to initiate fusion [17]. Moreover, structural analysis of immature CHIKV virus-like particles containing mutations in the furin cleavage site showed that E3 stabilizes domain B of E2 and prevents exposure of the fusion peptide on E1 [18]. However, for some alphaviruses (e.g., VEEV, SFV, and CHIKV), E3 may not fully dissociate from E2, which may depend in part on the pH of the medium in which the virus is produced [19–21]. Although the functional significance of retained E3 on the virion remains uncertain, it could impact receptor binding.

E2 is comprised of 3 principal ectodomains, A, B, and C [22,23]. A subdomain D within E2 also was identified in the VEEV crystal structure [19], seen in SINV [23], and contains key residues for SFV budding [24]. This subdomain has also been referred to as the E2 stem region [11]. Domain B is positioned furthest from the lipid bilayer, domain C is membrane proximal, and domain A is located between domains B and C [22]. E2 also contains a β-ribbon motif that connects domains A and B [11,22]. E1 is a class II fusion protein [25] that has 3 ectodomains, DI, DII, and DIII [26]. The hydrophobic fusion loop (FL) is located in DII [26]. E1 also contains a stem region that connects DIII to the transmembrane domain of the protein [11,26,27]. DIII adopts an immunoglobulin-like fold and is connected to DI through a linker region of approximately 28 amino acids [28].

Upon exposure to low pH in solution or in endosomes [6], E1 dissociates from E2 [29,30], which exposes the hydrophobic FL (Fig 1D). Subsequently, E1 forms a homotrimer, which triggers membrane fusion and enables nucleocapsid penetration into the cytosol. A computational study predicted that highly conserved histidine residues across 13 different alphavirus species located at the E1/E2 interface mediate the dissociation of E2 from E1 [31]. This model is consistent with structural [23] and biochemical analyses suggesting that conserved histidine residues stabilize E1/E2 interactions [32,33]. A recently reported 3.5 Å resolution cryo-electron microscopy (cryo-EM) structure of SINV corroborated observations from prior analyses [11,22] and provided new insights into the features governing the dissociation of E2 from E1. In addition to the role of conserved histidine residues, this study identified a novel hydrophobic pocket formed by E2 subdomain D and the E2 and E1 transmembrane helices [23]. Decreases in pH might disrupt this hydrophobic pocket, which, along with changes in hydrogen bonding between the conserved histidine residues, could destabilize E1/E2 interactions and promote E1 homotrimerization and membrane fusion [23].

The E1 DI and DII subdomains fold into a hairpin-like structure [11] following trimerization. Domain DIII packs against DI and DII [34] and participates in a “fold-back” mechanism that brings the viral envelope and target membranes in proximity [35]. E1-mediated membrane fusion is dynamic, with several intermediates described [22,36,37]. E1 initially engages the target membrane as 3 individual monomers, while E2 is still complexed as a trimer [36]. Using truncated forms of SFV E1 in vitro, a stable E1 trimer was shown to consist of only DI and DII at low pH (pH 5.7) in the absence of DIII or hairpin formation [36]. Thus, E1 trimerization likely occurs prior to the fold-back of DIII. Accordingly, exogenously expressed DIII can inhibit membrane fusion by acting as a dominant negative [38].

## Alphavirus attachment

For alphaviruses to initiate infection, they must attach to target cells and engage an entry receptor. Over the years, alphavirus interactions with several ubiquitously expressed cell surface molecules, termed attachment factors, have been described [39]. We distinguish between an attachment factor, which allows the virus to make initial contact with the target cell and entry receptors, which facilitate internalization of the virus prior to endosomal fusion [40]. Although a consensus definition is not established, we suggest a protein is a bona fide virus receptor if the following features are confirmed: (1) direct and specific binding interaction between virus and receptor; (2) the receptor directly mediates and/or facilitates internalization of the virus; (3) virus infection is blocked by antibodies against the receptor, by soluble receptor decoy molecules, or through mutagenesis of the virus receptor binding domain; and (4) susceptibility of a permissive cell type correlates with receptor expression level [41]. While many virus receptors are expressed on the cell surface [41,42], some viruses such as those in the *Filoviridae* family require engagement of an endosomal receptor (e.g., Niemann–Pick C1 (NPC1)) to successfully complete the viral entry process [43].

**Fig 2:**
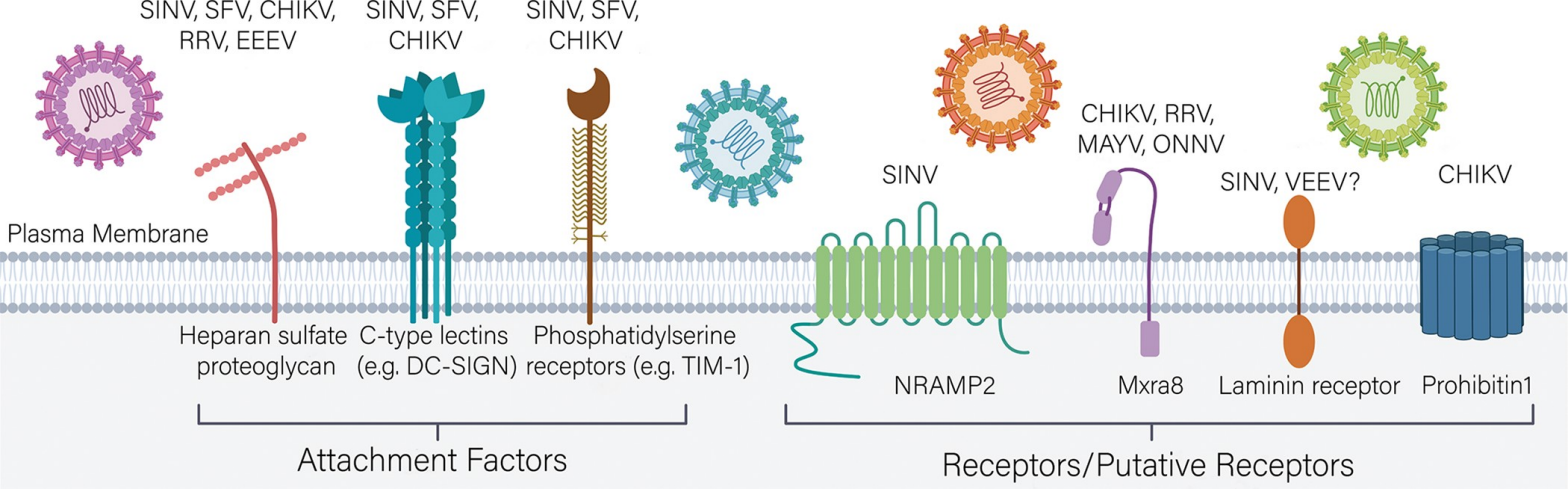
Alphavirus attachment factors entry receptors. Alphaviruses utilize several cell surface molecules including HS, DC-SIGN/L-SIGN, and PS receptors to carry out an initial attachment to target cells. These molecules increase infectivity of multiple alphaviruses and may also enhance virus internalization. Alphavirus receptors that satisfy all criteria to describe a protein as a virus receptor include NRAMP2 and Mxra8. Other putative receptors requiring further corroboration include laminin receptor and PHB1. CHIKV, Chikungunya; DC-SIGN, dendritic cell-specific intercellular adhesion molecule-3-grabbing non-integrin; EEEV, Eastern equine encephalitis virus; HS, heparan sulfate; L-SIGN, liver-specific SIGN; MAYV, Mayaro; Mxra8, matrix remodeling associated protein 8; NRAMP2, natural resistance-associated macrophage protein 2; ONNV, O'nyong-nyong; PHB1, prohibitin1; PS, phosphatidylserine; RRV, Ross River; SFV, Semliki Forest; SINV, Sindbis; TIM-1, T cell immunoglobulin mucin domain 1.

Attachment factors, in comparison, may display some but not all characteristics of receptors. However, virus binding and internalization generally are still observed in the absence of a given attachment factor, perhaps at a lower efficiency. Nevertheless, attachment factors can be important to viral pathogenesis, as they enhance target cell binding and decrease the amount of time the virus spends in the extracellular milieu, which, if prolonged, can lead to virus inactivation [44]. Below, we describe some of the best supported attachment factors and receptors for alphaviruses (Fig 2).

## Attachment factors

### Heparan sulfate

Several alphaviruses use heparan sulfate (HS), a negatively charged glycosaminoglycan (GAG), as an attachment factor either naturally or as an adaptation after passage in culture. A curious feature of HS-mediated attachment is that it can increase alphavirus infectivity in cultured cells but either increase or decrease virulence in vivo depending on the virus and route of inoculation [45–48]. Initially, HS expression was shown to increase the infectivity of SINV [49,50] in a manner dependent on the extent of sulfation [48]. SFV also bound liposomes containing heparin after frequent passage and adaptation of the virus [51]. A more recent genome-wide, exon-trap screen in human HAP-1 cells identified the enzyme N-deacetylase and N-sulfotransferase 1 (*NDST1*), which facilitates N-sulfation of HS, as critical for infectivity of a pseudotyped virus bearing the E1 and E2 glycoproteins of a clinical CHIKV isolate [52]. The reduced infectivity of the pseudotyped virus was not rescued by the addition of chondroitin sulfate (CS), another cell surface GAG, which suggested that the phenotype was HS specific. A separate CRISPR/Cas9 screen in HAP-1 cells also identified GAG biosynthesis genes including *B3GAT3*, *SLC35B2*, and *PAPSS1* as important factors for CHIKV infection [53].

The E2-82 residue in domain A of CHIKV E2 reportedly determines HS interactions. An arginine at this position (present in cell culture–adapted strains) results in enhanced HS interaction, and a glycine (present in clinical isolates) results in less binding to HS [54]. CHIKV strains containing the G82R mutation show reduced musculoskeletal disease and pathogenesis in mice [47]. The G82 residue also contributes to CHIKV persistence by allowing the virus to evade antibody neutralization and immune-mediated clearance [55].

The dependence of RRV on HS as an attachment factor was shown in the context of passage in avian cells [56] even though birds are not a reservoir species. Three mutations allowed RRV to replicate to higher titers [56]. Two of the variants had lysine substitutions at the surface-exposed residue E2-218 in domain B of E2 (N→K) [56], and a subsequent study showed that an arginine (N→R) at E2-218 also increased infectivity in an HS-dependent manner [57]. Cryo-EM experiments with the E2-N218R mutant of RRV revealed that HS binds the most distal portion of E2 [58,59]. In a separate study with an RRV E1 and E2 pseudotyped virus, a charge mutation to the adjacent residue E2-216 (T→R) also promoted the use of HS as an attachment factor [60]. As alluded to above, several HS-adapted arthritogenic alphaviruses show reduced virulence in vivo [48,54]. One explanation is that these virions become trapped by HS binding at the cell surface and bud less efficiently [60]. Alternatively, HS binding may target virus to cells that are inherently non- or less permissive and prevent productive spread [46].

Although the adaptation of HS binding for some alphaviruses leads to increased cell culture infectivity yet decreased pathogenicity in vivo, some natural alphavirus isolates use HS as a virulence factor in specific contexts. Non-passaged EEEV isolates use HS as an attachment factor [45]. Mutagenesis of 3 conserved lysine residues in domain A of E2 (E2-71, E2-74, and E2-77) abrogated EEEV binding to HS and decreased neurovirulence in mice [45]. Five naturally occurring EEEV strains with sequence variation at E2-71 and E2-72 showed differential dependence on HS as an attachment factor and distinct infectivity in mice [61]. EEEV strains that more readily bind HS are less pathogenic in mice after subcutaneous inoculation but display increased neurovirulence when delivered via intracranial injection [61]. Increased neurovirulence conferred by HS binding has also been reported with other neurotropic alphaviruses [62–64].

Initial cryo-EM analysis of HS analogs in complex with EEEV [61,65] suggested that the binding interface consists of the 3 key lysine residues at E2-71, E2-74, and E2-77, which are located within a surface-exposed β-strand and loop in E2 domain A [65]. However, a more recent, higher resolution 5.8 Å structure of EEEV in complex with HS showed that each spike contains 4 HS contact points, 3 along the quasi-3-fold axis of symmetry and 1 at the vertex of the spike [66]. While several basic amino acid residues were identified as part of the HS binding interface [66], the higher resolution structure did not identify the E2-71, E2-74, and E2-77 triad as directly interacting with HS. The basis for the disparity remains unclear, although the type of HS used might contribute to the differences [66].

Although the duration of viremia and magnitude of virus dissemination is reduced for HS-binding alphaviruses, the potentially deleterious effect on virus spread is counterbalanced by the enhanced neurovirulence phenotype conferred by adaptation to HS. HS-binding neurotropic alphavirus isolates that can bypass the requirement for sustained viremia to access the central nervous system may have a greater potential for virulence. Further studies are warranted to better understand the pathogenic mechanisms mediated by HS binding during neurotropic alphavirus infections including interactions at the blood–brain barrier [62]. More studies are also needed to distinguish HS adaptations that occur in cell culture from those present in natural isolates and how these sequence changes differentially impact alphavirus infection, tropism, immunity, and pathogenesis in mammalian and mosquito vector hosts.

### C-type lectins

C-type lectins, including dendritic cell-specific intercellular adhesion molecule-3-grabbing non-integrin (DC-SIGN) and liver-specific SIGN (L-SIGN), can act as an attachment factors for some alphaviruses [67,68]. In addition to their role in cell migration [69], these proteins function as pattern recognition receptors (PRRs) by binding high-mannose N-glycans on the surface of pathogens [70]. Alphaviruses, along with viruses from other families [71], can exploit this interaction to gain access to permissive cells. Cells transfected with either DC-SIGN or L-SIGN showed increases in SINV binding and infectivity [67]. Alphavirus binding to these lectins was observed only with virus generated in mosquito cells, which produce high- and pauci-mannose N-linked glycans compared to the complex N-linked glycans of vertebrates [72] and in mammalian cells lacking glycosyltransferases or treated with alpha-mannosidase inhibitors. These experiments demonstrated that differential mannose processing on N-linked glycans affects alphavirus interactions with DC-SIGN or L-SIGN. Consistent with these results, lentivirus pseudotyped with SFV E1 and E2 glycoproteins display enhanced transduction efficiencies in C-type lectin-expressing cells when the virus is generated under conditions that maintain high-mannose N-glycosylation [68]. Thus, high-mannose glycosylation of alphavirus glycoproteins can influence the tropism of target cells early during infection in mammalian hosts, which may impact the outcome of disease [73].

In addition to acting as alphavirus attachment factors, C-type lectins also function as PRRs. However, it is unclear whether their downstream signaling pathways are activated upon alphavirus engagement, which could impact post-entry steps in the alphavirus replication cycle [74]. C-type lectin signaling pathway activation could have functional implications for DC and/or macrophage maturation, including effects on major histocompatibility complex (MHC) class I presentation, adaptive immune responses, and pathogenesis [70]. Indeed, particle size and structure can dictate how antigens are internalized by DCs [75]. Upon binding to DC-SIGN, smaller, polymeric structures are routed to endosomes, whereas larger structures characteristic of viruses localize to non-endosomal compartments [75]. It remains to be determined how engagement of DC-SIGN and other C-type lectins affects the subcellular trafficking and infectivity of alphaviruses. Despite their effects on infectivity in cell culture, studies in mice deficient in DC-SIGN or L-SIGN expression did not show an impact on CHIKV pathogenesis [76]. In comparison, expression of the C-type lectin dendritic cell immunoreceptor (DCIR) limited CHIKV disease, an effect which may require direct binding of CHIKV [76]. The physiological significance of interactions with DC-SIGN/L-SIGN and other C-type lectin receptors on disease progression and immunity with other arthritogenic or encephalitic alphaviruses is undetermined.

### Phosphatidylserine receptors

Phosphatidylserine (PS) is a component of the eukaryotic plasma membrane that is found in the host-derived lipid bilayer of many enveloped viruses [77]. PS receptors are increasingly recognized as attachment factors and/or receptors for viruses [77]. The most well-characterized function of PS receptors is the binding of surface-exposed PS during apoptosis and subsequent signaling of dying cells to be marked for phagocytosis [78]. Accordingly, this has led the viral hijacking of PS receptors to be termed “apoptotic mimicry” [79]. The T cell immunoglobulin mucin (TIM) domain family proteins were the first PS-binding receptors proposed as attachment factors for SINV, CHIKV, RRV, and EEEV [80,81]. Infection of pseudotyped viruses displaying the alphavirus E1/E2 glycoproteins was increased in cells expressing TIM-1 and decreased in the presence of PS-containing membranes, suggesting that infectivity depends on TIM-1 binding of PS as opposed to a direct interaction with the virus glycoproteins [80]. Ectopic expression of TIM-1 also increased RRV uptake and infection in cells, and this phenotype was blocked by incubation with an anti-TIM-1 antibody [80]. A separate study using pseudotyped viruses expressing SINV envelope proteins [82] expanded the list of PS-binding proteins used by alphaviruses as attachment factors to include milk fat globule-epidermal growth factor-factor 8 (MFG-E8) and growth arrest-specific gene 6 (Gas6), 2 soluble adaptor molecules that engage PS [82]. Another PS receptor, CD300a, also increased the binding of pseudotyped virus displaying SINV E1/E2 to cells but did not enhance infection rates [82]. The effect of PS receptor engagement by alphaviruses on apoptosis signaling pathways has not been extensively investigated, which independently may impact immunity and pathogenesis.

Many questions remain about the interactions between attachment factors and receptors in alphavirus entry. Some groups have speculated that an exceptionally high affinity virus receptor could preclude a requirement for PS binding or other attachment factors [81,83]. Thus, alphaviruses with lower affinities for their viral receptor might preferentially use other attachment factors. For most alphavirus attachment factors, the full picture of how they function to enhance viral infection in vivo is incomplete.

## Receptors

The identification of bona fide receptors for alphaviruses historically has been elusive. One impediment has been the lack of a discernable interaction between putative receptors and purified E2 proteins [84]. Moreover, for many proposed receptor molecules, infection has still been shown to occur in cells lacking the protein, suggesting that either receptor usage is highly cell-type–specific and/or that the proposed molecule is a subordinate receptor on most cells or acts more as an attachment factor to enhance infectivity. In the following section, we describe recent progress on alphavirus receptors including different proteins with their varying degrees of supportive data.

### NRAMP

Natural resistance-associated macrophage protein (NRAMP) proteins are divalent metal ion transporters that have been proposed as receptors for SINV in both insect and mammalian cells [85]. Gene silencing of dNRAMP (the *Drosophila* gene) resulted in decreased infection for both cell culture-adapted and wild-type SINV strains in fruit flies [86]. As transfection of SINV RNA directly into *Drosophila* cells bypassed a requirement for dNRAMP, this protein was hypothesized to function during alphavirus entry. Indeed, direct virus binding to and interaction with dNRAMP was demonstrated by co-immunoprecipitation and confocal microscopy assays. The mammalian NRAMP2 protein is ubiquitously expressed on the cell surface of neuronal cells and macrophages [85]. SINV infection also was reduced in NRAMP2-deleted mouse embryonic fibroblasts (MEFs) and in several mammalian (MEF and U2OS) and insect (*Drosophila* DL1 and *Aedes aegypti* Ag-2) cell lines treated with exogenous iron, which downregulates dNRAMP/NRAMP2 protein expression. In contrast, infection of a chimeric alphavirus displaying the RRV envelope proteins was insensitive both to iron treatment and to NRAMP2 deletion. It is unclear whether other alphaviruses use NRAMP2 or other conserved membrane transporter proteins as receptors for infection. It also will be important to determine the role of NRAMP2 in SINV pathogenesis in vivo, as this has not yet been tested. Finally, alphavirus infection studies with blocking antibodies against NRAMP2 or receptor decoy molecules could provide further evidence for this protein as a bona vide receptor.

### Mxra8

In a genome-wide CRISPR/Cas9 screen, matrix remodeling associated protein 8 (Mxra8) was identified as a receptor for several arthritogenic alphaviruses, including CHIKV, RRV, MAYV, and ONNV [87]. Mxra8 is expressed on the surface of epithelial, mesenchymal, and myeloid cells [87], all of which are targets of infection by arthritogenic alphaviruses [88]. Several lines of evidence support Mxra8 as an alphavirus receptor: (1) ectopic expression of Mxra8 enhances alphavirus infection; (2) transfection of viral RNA into cells bypasses a requirement for Mxra8 expression; (3) CHIKV binding to and infection of cells is blocked with antibodies against Mxra8 or a soluble Mxra8-Fc decoy protein; and (4) Mxra8 binds directly to CHIKV viruses or virus-like particles by enzyme-linked immunosorbent assay (ELISA) and surface plasmon resonance. Mxra8 also is required for arthritogenic alphavirus pathogenesis, as infection in vivo was inhibited with blocking Mxra8-Fc treatment, anti-Mxra8 antibodies, or in Mxra8-deficient mice [87,89]. However, low levels of viral infection occurred in cell culture and in vivo in the absence of Mxra8 expression, suggesting the existence of an unidentified subordinate receptor for this group of viruses. Outstanding questions remain on how Mra8 facilitates alphavirus internalization. Given that the cytoplasmic tail of Mxra8 is not required for receptor function [87], it is possible that Mxra8 engagement triggers interaction with an unidentified co-receptor that activates the endocytic pathway.

**Fig 3:**
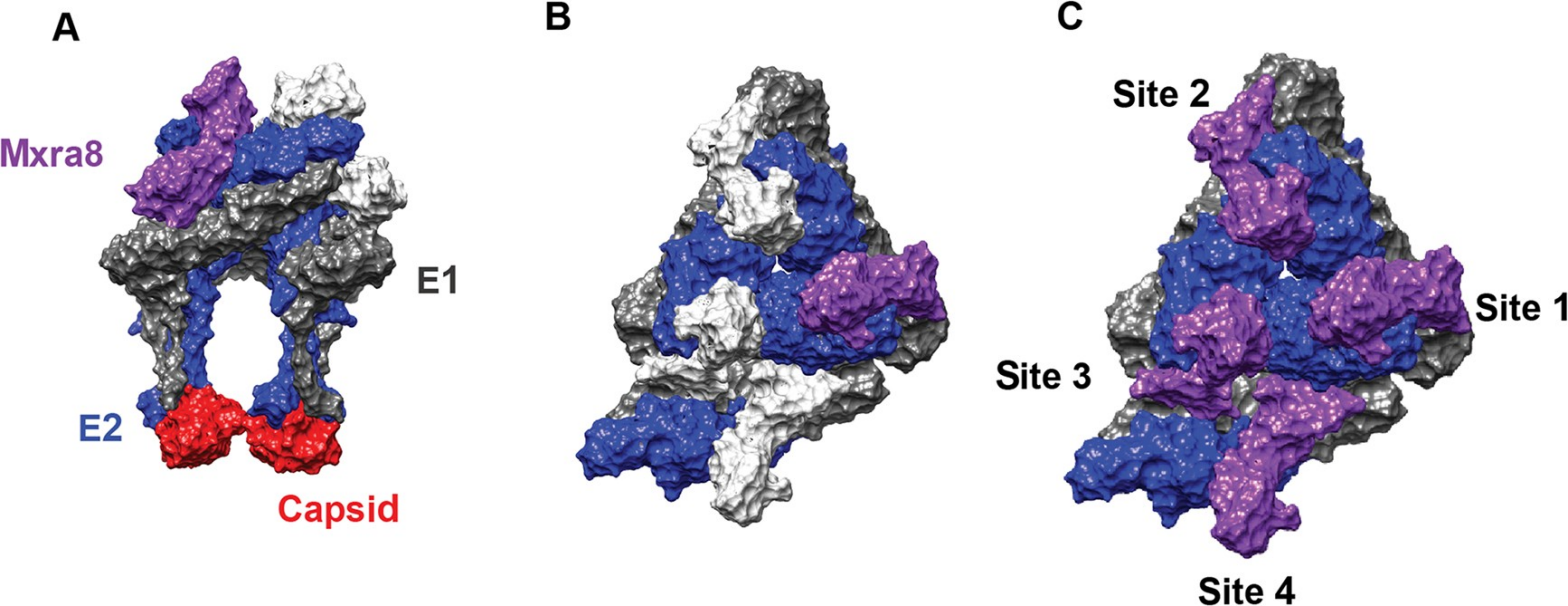
Mxra8 binding to CHIKV E1 and E2. Side view **(A)** and top view **(B and C)** of Mxra8 bound to E1 and E2. Mxra8 (purple) wraps around 1 E1 (gray)-E2 (blue) heterodimer, contacts a second heterodimer within the same spike, and makes contacts with a third heterodimer on the adjacent spike. Capsid proteins are pictured in red. Mxra8 engages the CHIKV spike structure in a complex quaternary epitope. Image generated with Chimera software using [21] as a reference (PDB: 6NK6). CHIKV, Chikungunya virus; Mxra8, matrix remodeling associated protein 8.

Structural analysis of Mxra8 in complex with either CHIKV virions [21] or E1/E2 glycoprotein complex [90] was recently described. Mxra8 ectodomain is comprised of 2 Ig-like domains arranged in an unusual head-to-head configuration [21,90]. A single monomer of Mxra8 can engage 3 different E1/E2 heterodimers on the virion surface [21,90]. Mxra8 wraps around the membrane-distal end of 1 E1/E2 dimer, makes intraspike contacts with a second heterodimer, and engages a third neighboring E1/E2 complex in an interspike interaction [21,90] (Fig 3A–3C). Mxra8 contacts residues in domains A and B of E2 and the FL and other sites in E1 DII. The binding occupancy of Mxra8 to infectious CHIKV particles is reduced by the presence of E3 on the virion [21]. Thus, retention of bound E3 even after cleavage and maturation could affect alphavirus binding and Mxra8 usage as a receptor. Future studies should determine whether the presence of E3 on some arthritogenic alphaviruses (e.g., SFV) explains their relatively weak dependence on Mxra8 for binding.

Evolutionary and functional analyses established that most members of the *Bovinae* subfamily have a 15-amino-acid insertion in the Mxra8 ectodomain that blocks CHIKV binding. Introduction of this sequence into murine Mxra8 abolishes binding to virus particles and reduces CHIKV pathogenesis in vivo, whereas removal of the insertion in *Bovinae* Mxra8 enhances binding and infection [86]. This insertion likely evolved approximately 5 million years ago in the Miocene epoch [86], which could suggest that sequence acquisition was driven by positive selection against a primordial alphavirus. As alphaviruses are believed to have evolved much more recently (approximately 10,000 years ago) [91], this idea remains speculative.

### Laminin receptor

Based on monoclonal antibody blocking studies, the laminin receptor was proposed as a possible receptor for SINV [92]. Laminin receptor is a cell surface-expressed protein that binds basement membrane laminin and has functions in both development and tumor metastasis [92]. SINV binds Chinese hamster ovary (CHO) cells overexpressing laminin receptor more readily than the parental control cell line, and the susceptibility of BHK cells increases with the amount of laminin receptor expressed on the cell surface [92]. Together with experiments showing inhibition of SINV infection by antibodies against laminin receptor, 3 of the criteria are met for establishing laminin receptor as a receptor. However, laminin receptor-mediated internalization of SINV (or any other alphavirus) or direct biochemical binding has yet to be documented. While a more recent study suggested that the carboxyl-terminal domain of laminin receptor interacts with VEEV E2 [93], an interaction with this encephalitic alphavirus has not been critically evaluated.

### PHB1

Prohibitin1 (PHB1) is a protein that localizes to the plasma membrane and mitochondria and regulates cell proliferation and mitochondrial integrity [94]. One paper has described PHB1 as a receptor for CHIKV [95]. Using a proteomic approach, PHB1 was identified as a candidate binding partner for 2 CHIKV strains. Incubation of a microglia cell line with increasing concentrations of an anti-PHB1 antibody decreased the percentage of CHIKV-infected cells and viral yield. This observation was supported by gene silencing of PHB1, albeit this resulted in a relatively small reduction in CHIKV virus production. PHB1 also co-immunoprecipitated with CHIKV E2 and co-localized with CHIKV E2 at the plasma membrane by immunofluorescence microscopy. Although these data suggest that PHB1 might act as a receptor for CHIKV, no direct binding of CHIKV virions with soluble PHB1 has been demonstrated, and the effects of PHB1 on virus binding to and internalization in cells have not been reported. Of note, PHB1 reportedly also interacts with Dengue virus, a Flavivirus, to facilitate entry into insect cells [96].

## Cellular uptake mechanisms of alphaviruses

Alphaviruses are internalized principally by clathrin-mediated endocytosis and delivered to the endosomal compartment where membrane fusion occurs, a process that has been reviewed extensively by others [39,97–99]. Live video tracking studies have shown that most CHIKV particles co-localize with clathrin prior to undergoing fusion [100]. A selective inhibitor of clathrin-mediated endocytosis, Pitstop, substantially reduced the number of CHIKV-infected cells, indicating a strong dependence on this entry pathway. CHIKV fusion primarily occurs in early endosomes as indicated by co-localization of virus particles with Rab5, a marker of this compartment [100]. Consistent with these data, a genome-wide RNA interference (RNAi) screen using SINV identified other host proteins important for clathrin-mediated endocytosis, including Fuzzy homologue (FUZ) and the tetraspanin membrane protein, TSPAN9 [101], which was specifically important for low-pH-triggered membrane fusion in the early endosome [101].

In some studies, alphaviruses are reported to internalize via alternate pathways including caveolae-dependent entry of MAYV into Vero cells [102], the direct delivery of SINV to target cells through a putative pore at the plasma membrane [103], and micropinocytosis-mediated uptake of CHIKV into muscle cells [104]. Considering the discovery of novel entry receptors for alphaviruses [85,87], and as yet unknown alternative receptor(s), it will be important to determine whether engagement by specific moieties on the surface facilitates distinct entry pathways of different alphaviruses in unique cell types.

### Therapeutics targeting alphavirus cell entry

Currently, there are no Food and Drug Administration (FDA)-approved vaccines or antiviral drugs for pathogenic alphaviruses. Given that virus entry is the first step required to initiate a productive infection and can require highly specific interactions with receptors, it is an attractive target for the development of alphavirus antivirals.

**Fig 4:**
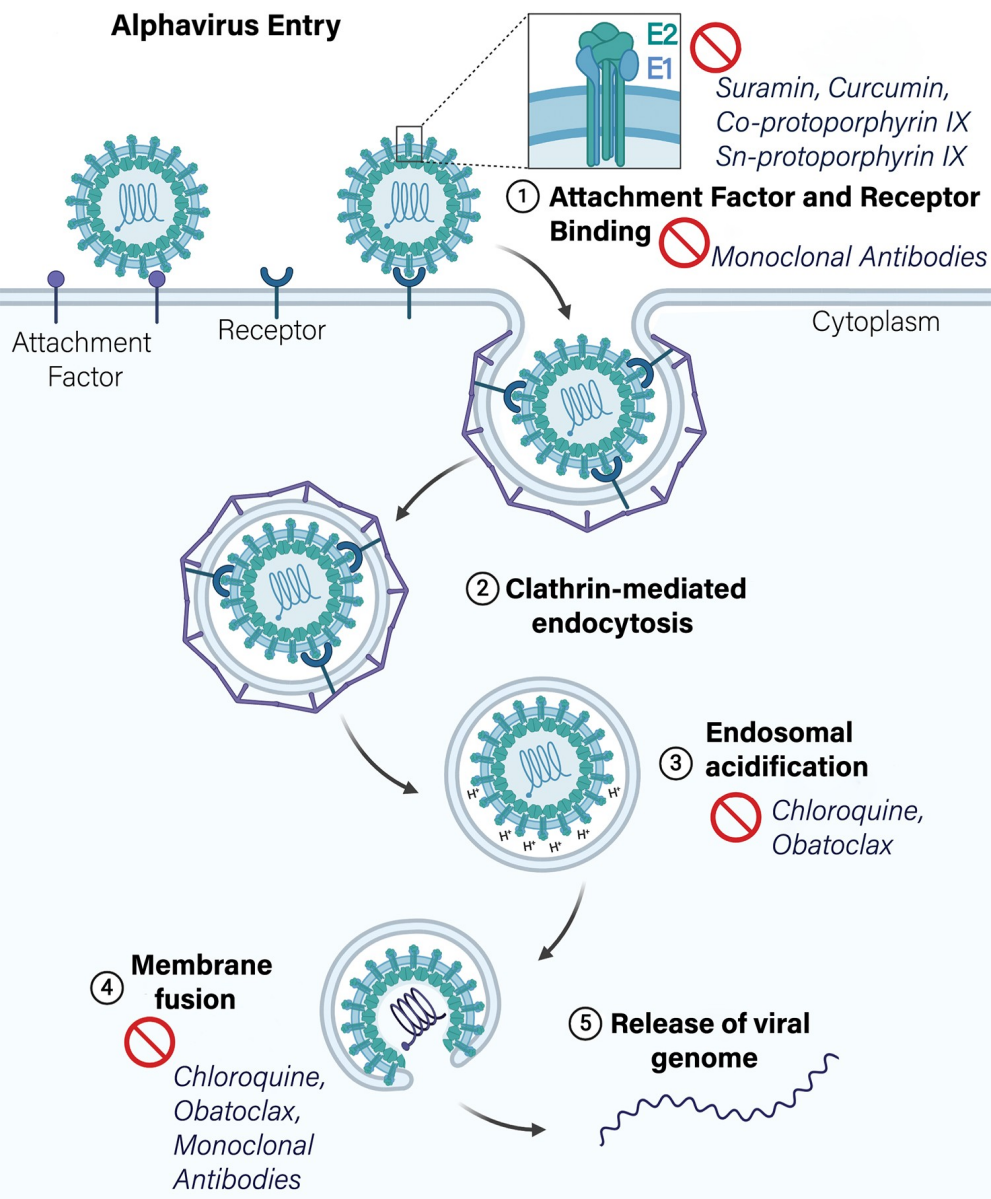
Schematic diagram of alphavirus entry pathway and inhibitors targeting each step. Inhibitors that target alphavirus attachment and receptor binding, endosomal acidification, membrane fusion, and E1/E2 stability.

Candidate therapeutics that target alphavirus attachment and entry have recently been described (Fig 4). These include neutralizing monoclonal antibodies that block attachment, internalization, and pH-dependent fusion [105–107]. Monoclonal antibodies can be developed quickly during epidemics [107], have a rapid onset of protection [107], and facilitate viral clearance through multiple mechanisms including direct neutralization of virus [108] and indirect antibody-dependent effector functions including cell-mediated cytotoxicity [107,109], complement-dependent cytotoxicity, and phagocytosis [107]. A phase I/II clinical trial (NCT02230163) was initiated to determine the safety and efficacy of anti-CHIKV hyperimmune sera for treating neonatal infections resulting from vertical CHIKV transmission [110]. Another phase I trial was initiated to test the safety and tolerability of lipid-encapsidated mRNA encoding a neutralizing anti-CHIKV monoclonal antibody [111] (NCT03829384). Administration of the anti-inflammatory drug, CTLA4-Ig (also known as Abatacept), in conjunction with a neutralizing monoclonal antibody against CHIKV was highly protective against virus pathogenesis in mice [112]. The combination of antiviral and anti-inflammatory therapy may be a promising strategy for treating arthritogenic alphavirus infections and the ensuing immunopathology [113].

Other studies have identified small molecule inhibitors of alphavirus entry (Table 2) [114–116]. These include suramin, a known antiparasitic drug [117]. Molecular docking studies predict suramin intercalates between E1 DII and E2 domain C [114], potentially disrupting the stability of the E1/E2 heterotrimer and, in turn, its associated entry functions. Suramin also has activity against CHIKV in vivo and reduces virus burden and foot swelling in mice [118]. Other compounds that affect alphavirus entry include curcumin [116], a naturally occurring phenol, which reduces infectivity and blocks cell binding of CHIKV and Zika virus (ZIKV), an unrelated flavivirus [116]. Accordingly, transfection of viral RNA into cells in the presence of the drug bypasses the antiviral activity [116]. The flavonoid compound baicalin is also believed to affect CHIKV entry steps [119], although the exact mechanism of action is unknown. Co-protoporphyrin IX and Sn-protoporphyrin IX, 2 porphyrins that affect virus envelope integrity, impair adsorption of CHIKV, MAYV, SFV, and SINV to cells [120]. Obatoclax, another broad-spectrum antiviral against enveloped viruses, inhibits CHIKV and SFV infection by neutralizing endosomal pH and inhibiting fusion [121]. Chloroquine, a well-characterized antimalarial drug, has efficacy against CHIKV in cultured cells by neutralization of endosomal pH [122,123]. Although effective in cultured cells, studies in nonhuman primates showed that chloroquine treatment paradoxically resulted in higher viremia and delayed viral clearance [124], an effect that correlated with altered type I IFN responses. In human infections treated with chloroquine, viremia and persistent polyarthralgia were not improved [124]. Overall, these results with chloroquine highlight a need for caution when extrapolating from cell culture to in vivo systems.

**Table 2:** Inhibitors of alphavirus entry.

Therapeutic target	Entry inhibitor [reference]	Mechanism of action
1. Attachment factor and receptor binding	Monoclonal antibodies [107]; curcumin [116] Co-protoporphyrin IX and Sn-protoporphyrin IX [120] Suramin [114,115,118,142–144]	Block receptor binding, ADCC; CDC Engage in hydrophobic interactions in the lipid bilayer to disrupt envelope integrity E1/E2 heterotrimer, may disrupt heterodimer assembly
2. Clathrin-mediated endocytosis	Pitstop [145]	Clathrin inhibitor
3. Endosomal acidification	Obatoclax [121], chloroquine [122–124]	Neutralizes endosomal pH
4. Membrane fusion	Co-protoporphyrin IX and Sn-protoporphyrin IX [120] Monoclonal antibodies [107]	Disrupt viral envelope integrity; block fusion

Therapeutics that disrupt alphavirus entry include monoclonal antibodies and small molecule inhibitors. Steps in the entry pathway targeted (1) receptor binding, (2) clathrin-mediated endocytosis, (3) endosomal acidification, and (4) membrane fusion. ADCC, antibody-dependent cellular cytotoxicity; CDC, complement-dependent cytotoxicity.

Novel broad-spectrum antivirals that target fusion and internalization of several enveloped viruses including the alphavirus SFV were recently identified [125]. While these compounds reduced viral burden in cell culture and in vivo, they did not improve survival, at least in a mouse model of ZIKV infection. Moreover, inhibitors of entry that affect endosomal pH could have off-target effects given that this is a critical pathway in cellular homeostasis. The most effective entry inhibitors should block the attachment step, as this requires a highly virus-specific interaction between the receptor and viral attachment proteins. Indeed, most clinically approved entry inhibitors focus on this aspect of the life cycle [126] for viruses including human immunodeficiency virus (HIV), respiratory syncytial virus, varicella-zoster virus, herpes simplex virus, and hepatitis C virus. Other broad-spectrum antivirals that target the viral entry step include the envelope intercalating agent, LJ1001, which exploits differences in the biophysical properties of the viral and host membranes to prevent viral fusion while leaving host membranes unaffected [127].

## Conclusions

Alphaviruses are rapidly emerging and reemerging human pathogens. Important structural, biochemical, and molecular insights into the entry step of the alphavirus life cycle have been made. These advances have enhanced our understanding of how alphaviruses attach to and invade target cells, how entry influences tissue tropism and virus pathogenesis, and importantly, highlight facets of the entry process for targeting with antiviral therapeutics. However, several key questions remain: What factors drive adaptation to HS attachment factor usage in cell culture versus natural adaptation? What are the alternative receptors for arthritogenic alphaviruses? What are the attachment and entry receptors for encephalitic alphaviruses? What are the entry pathways utilized by the currently described receptors for alphaviruses? How often and in what situations do non-clathrin-mediated entry pathways occur? How does the entry pathway affect tropism and immune evasion? How does receptor and/or attachment factor usage and their downstream signaling pathways contribute to alphavirus pathogenesis in vivo? Answering these fundamental questions will address gaps in our knowledge of the alphavirus entry pathway and may allow for the generation of countermeasures that more precisely target this critical first step in the alphavirus infection cycle.
